# Modulation efficiency of clove oil nano-emulsion against genotoxic, oxidative stress, and histological injuries induced via titanium dioxide nanoparticles in mice

**DOI:** 10.1038/s41598-024-57728-1

**Published:** 2024-04-02

**Authors:** Hanan R. H. Mohamed, Sawsan El-Shamy, Sherein S. Abdelgayed, Rofida Albash, Haidan El-Shorbagy

**Affiliations:** 1 Zoology Department, Faculty of Science, Cairo University, Giza, Egypt; 2 Faculty of Biotechnology, October University for Modern Science and Arts, 6th October, Giza Egypt; 3 Pathology Department, Faculty of Veterinary Medicine Cairo University Giza, Giza, Egypt; 4 College of Oral and Dental Surgery, Misr University for Science and Technology, 6th of October, Giza Egypt; 5 Department of Pharmaceutics, Faculty of Pharmaceutical Sciences, Misr University for Science and Technology, 6th of October, Giza Egypt

**Keywords:** Titanium dioxide nanoparticle, Clove oil nanoemulsion, Titanium dioxide nanoparticles toxicity, Oxidative stress, Natural hazards, Risk factors

## Abstract

Titanium dioxide nanoparticles (TiO_2_-NPs) have found wide applications in medical and industrial fields. However, the toxic effect of various tissues is still under study. In this study, we evaluated the toxic effect of TiO_2_-NP on stomach, liver, and kidney tissues and the amelioration effect of clove oil nanoemulsion (CLV-NE) against DNA damage, oxidative stress, pathological changes, and the apoptotic effect of TiO_2_-NPs. Four groups of male mice were subjected to oral treatment for five consecutive days including, the control group, the group treated with TiO_2_-NPs (50 mg/kg), the group treated with (CLV-NE) (5% of the MTD), and the group treated with TiO_2_-NPs plus CLV-NE. The results revealed that the treatment with TiO_2_-NPs significantly caused DNA damage in the liver, stomach, and kidney tissues due to increased ROS as indicated by the reduction of the antioxidant activity of SOD and Gpx and increased MDA level. Further, abnormal histological signs and apoptotic effect confirmed by the significant elevation of p53 expression were reported after TiO_2_-NPs administration. The present data reported a significant improvement in the previous parameters after treatment with CLV-NE. These results showed the collaborative effect of the oils and the extra role of nanoemulsion in enhancing antioxidant effectiveness that enhances its disperse-ability and further promotes its controlled release. One could conclude that CLV-NE is safe and can be used as a powerful antioxidative agent to assess the toxic effects of the acute use of TiO_2_-NPs.

## Introduction

The versatility of TiO_2_-NPs in many care products and commercial applications increases the possibility of human exposure to TiO_2_-NP during the manufacturing and processing of the nanomaterial and via certain consumers’ goods (eg, sunscreens, enamels, varnishes, pigments for paint)^1^. The small size of TO_2_-NPs enables their uptake by cells and their transcytosis across the epithelium and endothelium cells, and their passage to the circulatory system to reach many target tissues, accordingly, their accumulation induced many inflammatory and toxic effects^2,3^. They increase the phagocytic capability of macrophages which provoke membrane and ultrastructure destruction and dysfunction of the liver and kidney^4–6^. This may highlight the possibility of changing the architecture of many vital organs like the kidney, liver, and lungs due to exposure to TiO_2_-NPs^7,8^. Moreover, TiO_2_-NPs can induce oxidative stress which raises the induction of lipid peroxidation that promotes genotoxicity and apoptosis within liver tissue^3,9,10^.

Cloves (*Syzygium aromaticum L.*) belongs to the *Myrtaceae* family, from which clove essential oil (CEO) is extracted from their flower buds and leaves. CEO contains many phenolic compounds, and eugenol is the major compound (85–95%)^11^. Eugenol has proven its efficiency as an antioxidant agent in many researches^12,13^. Further, eugenol revealed an anticancer activity against the human adenocarcinoma cell line in the lung^14^. Recently, many other biological effects of eugenol like anti-proliferative, anti-inflammatory, anti-microbial, and DNA damage protective effects have been reported^15–20^.

Nanoemulsion is composed mostly of an essential oil emulsion system composed of an average size droplet size of 200 nm. These oils mostly suffer from solubility differences between droplets size due to different radius curvature which is called Ostwald ripening (OR)^21^. Thus, nanoemulsion is mainly used to overcome the solubility problem and to increase the surface area of the oil. Therefore, the present study aimed to assess the attenuation ability of CLV-NE against genotoxic, cytotoxic, histopathological, and biochemical effects detected after the administration of TiO_2_-NPs.

## Material and methods

### Animals

Thirty 6-week-old Swiss webster mice weighing 25–30 g were obtained from the National Organization for Drug Control and Research (Giza Egypt) and kept in the animal house of the Zoology Department, Faculty of Science Cairo University under standard housing conditions and given food and water ad libitum. Mice were fed completely nutritional diet called Mazuri's vegetarian rat and mouse diet manufuctured by Land O' Lakes Inc Company (Arden Hills, Minnesota, USA).

### Ethical consideration

This study was reported according to ARRIVE guidelines and all experiments of this study were and conducted according to the standard international guidelines for the care and use of laboratory animals and were approved by October University for Modern Sciences and Arts, Faculty of biotechnology Committee (Egypt). Animals handling and experimentations were also conducted in accordance with the Guidelines of the National Institutes of Health (NIH) regarding the care and use of animals for experimental procedures.

### Chemicals

Clove nano-emulsion was purchased from Naqaa Company for nanotechnology (Giza Egypt) with a concentration of 12–15%, biochemical kits were obtained from Bio-diagnostic Company (Giza, Egypt), while all other chemicals were purchased from Sigma-Aldrich (St. Louis, MO, USA) with high analytical grade.

### Characterization of TiO_2_-NPs

A mixture of rutile and anatase-shaped TiO_2_-NPs were purchased from Sigma-Aldrich (St. Louis, MO, USA) with purity of 99.5% and CAS number 13463-67-7. Prior to administration, the unscented white powders of TiO_2_-NPs were sonicated in deionized distilled water using an ultrasonic homogenizer (Model 150VT) and have been well characterized using transmission electron microscope, X-ray diffraction and Zeta Sizer^22^.

### Characterization of the clove oil nanoemulsion

The appearance and morphology of CLV-NE were studied as described by El-Shamy^23^, using transmission electron microscopy (TEM). One drop of CLV-NE was placed on a carbon laminated copper grid, then spread and stained using phosphotungstic acid 1.5%.

### Determination of maximum tolerated dose (MTD)

Acute toxicity assay was done to detect the maximum tolerated dose of CLV-NE using OECD guidelines (Guideline 2001). Five male mice were orally given clove nano-emulsion at a dose level of 2000 mg/kg according to the main test of OECD/OCDE. All mice were then carefully observed for 24 h after CEO nano-emulsion administration for any sign of toxicity, morphological behavior, and mortality. The studied doses of clove nano-emulsion were estimated as 5% of the safety dose obtained from the OECD test.

### Experimental design

Twenty male mice were randomly divided into four groups, five mice per group; mice of the negative control group were orally given deionized distilled water, while mice of the other three remaining groups were orally administered CLV-NE at a dose of 5% of the MTD or/and TiO_2_-NPs at a dose of 50 mg/kg^22^ for five consecutive days. After 24 h of the last administration, mice of the four groups were sacrificed and dissected to obtain stomach, liver, and kidney tissues. Part of these tissues was kept in 10% formalin for histological studies and other part was kept at − 80 °C for molecular and biochemical examination.

### Molecular studies

#### Laddered DNA fragmentation assay

The integrity of genomic DNA in stomach tissues of four groups was studied using Laddered DNA fragmentation assay^24^; small part of stomach tissues was gently homogenized in TE lysis buffer and incubated with RNase A at 37 °C for cells lysis. Proteinase K was then added, and all samples were incubated at 50 °C overnight. Whole genomic DNA was extracted and precipitated by cold absolute ethanol. The extracted genomic DNA was electrophoresed on 1% agarose gel at 70 V, visualized and photographed.

#### Expression of p53 gene

The whole gastric RNA was extracted using the GeneJET RNA Purification Kit (Thermo scientific, USA). The purity and concentration of the extracted gastric RNA were detected by a Nanodrop device. The extracted gastric total RNA was then reverse transcribed into complementary DNA (cDNA) using the Revert Aid First Strand cDNA Synthesis Kit (Thermo scientific, USA). Finally, quantitative RT-PCR reaction was conducted to amplify gastric p53 gene through the 7500 Fast system (Applied Biosystem 7500, Clinilab, Egypt) using 2 × SYBR Green Master Mix (Thermo Scientific, USA) and the primers sequences listed in Table 1^25^. The mRNA expression level of the p53 gene was standardized against expression of β actin housekeeping gene. The comparative *Ct* (ΔΔ*Ct*) method was used to quantify the expression levels of p53 gene and the results were expressed as mean ± S.D.

**Table 1:** Sequences of the Primers used in qRT-PCR.

Gene	Strand	Sequence
P53	Forward Reverse	5′-TACTCTCCTCCCCTCAATAAG-3' 5′- ACCATCGGAGCAGCCCTCAT-3'
β-actin	Forward Reverse	5′-TCACCCACACTG TGCCCATCT ACG A-3' 5′-GGATGCCACAGGATTCCATACCCA-3'

#### Biochemical analysis

Parts of Stomach, liver and kidney tissues were homogenized in 0.1 M phosphate buffer (pH 7.4) containing 1 mM EDTA, then centrifuged at 4000 rpm for 15 min in a cooling centrifuge (Sigma, D-37520 Osterode. Am Harz., Germany, Model 2–16 K), and the supernatant was pipetted into plastic tubes for determination of SOD and GpX as antioxidants according to the method of^26^ and^27^, respectively. According to^28^ method, MDA level was determined using Bio diagnostic kit following the instruction’ protocol where the absorbance of the resultant pink product can be measured at wave length 534 nm.

#### Histological studies

For histopathological studies, fresh portions of the stomach, liver and kidney tissues were fixed in 10% buffered formalin immediately before being embedded in paraffin wax, then they were sectioned into 5 µm thickness using microtome for staining using hematoxylin and eosin. For investigation and analysis, the stained sections were photographed under light microscope.

#### Statistical analysis

The Social Science Statistical Package (SPSS 23) was used to analyze the results obtained. A one-way analysis of variance (ANOVA) was performed to compare the effects of different treatments on the parameters studied. Duncan's test was performed to compare study groups. Data were expressed as mean ± standard deviation (SD).

## Results

### Characterization of clove oil nanoemulsion

**Figure 1:**
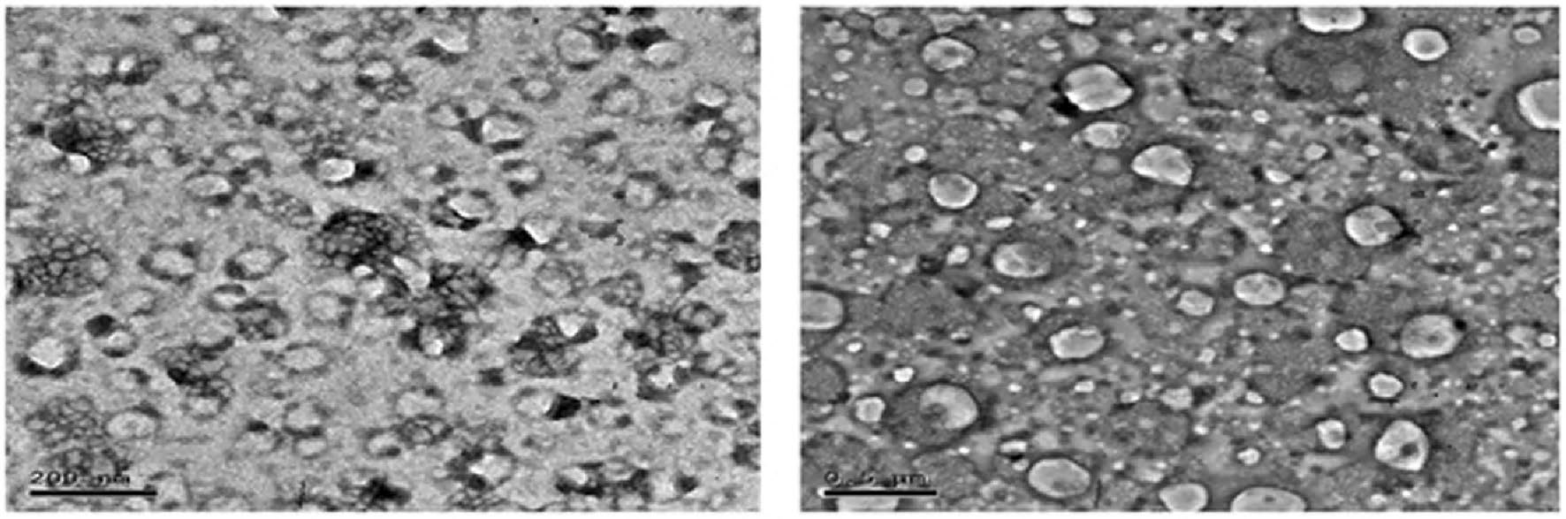
TEM photographs of clove oil nanoemulsion^23^.

Assessment of the external morphology of the CLV nanoemulsion by TEM^23^ revealed that they had a uniform size distribution measured at 218.1 ± 2.27 nm and a spherical shape (Fig. 1).

### MTD and tested dose of CLV-NE

Surveillance of mice orally given CLV-NE at the 2000 mg/kg dose level revealed that all mice were still healthy and no signs of toxicity were observed during the first 48 h of CLV-NE administration until the end of the 14-days monitoring period. Thus, the MTD and the half lethality dose (LD50) of CLV-NE was considered above 2000 mg/kg according to the OECD guidelines, and the initial tested dose of CLV-NE in this study was calculated as 5% (100 mg/kg body weight) of the obtained LD50 from acute toxicity test.

### Molecular studies

#### Laddered DNA fragmentation

**Figure 2:**
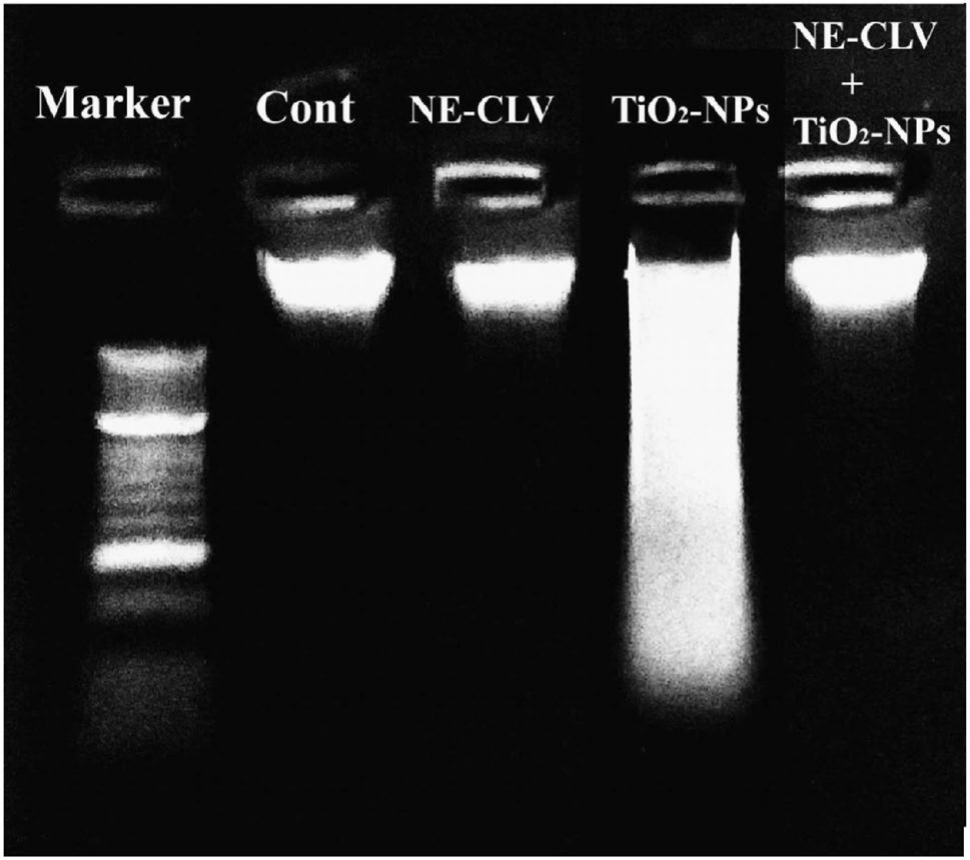
Pattern of the electrophoresed genomic DNA of the negative control group and mice administered clove nano-emulsion (CLV-NE) or/and TiO_2_-NPs.

As shown in Fig. 2 TiO_2_-NPs caused dramatic damage to genomic DNA as manifested by the observed smeared and fragmented DNA on agarose gel in comparison to the intact pattern of control genomic DNA. However, oral coadministration of CLV-NE with TiO_2_-NPs declined TiO_2_-NPs induced DNA damage and restored the integrity of genomic DNA as shown by the re-appearance of an intact genomic DNA pattern of mice given TiO_2_-NPs with CLV-NE (Fig. 2 & Fig. S1). Moreover, oral administration of CLV-NE does not cause any changes in the pattern of genomic DNA compared to that of negative control mice.

#### Expression of p53 gene

As shown in Table 2 oral co-administration of CLV-NE simultaneously with TiO_2_-NPs (1.16 ± 0.08^a^) caused statistically significant (*P* < 0.001) decrease in the expression level of p53 gene compared to their highly upregulated expression level in mice orally given TiO_2_-NPs alone (2.93 ± 0.59^b^) and even reached the negative control level. On the other hand, no significant differences were observed in the p53 expression level after administration of CLV-NE contrasted to the negative control (Table 2).

**Table 2:** p53 gene expression levels in the stomach tissues in negative control group and groups administered either by clove nano-emulsion or/and TiO_2_-NPs.

Group	Treatment (dose mg/kg)	P53
Negative control	0	1.00 ± 0.00^a^
CLV-NE	100 mg	1.11 ± 0.01^a^
TiO_2-_NPs	50 mg	2.93 ± 0.59^b^
CLV-NE + TiO_2_-NPs	100 mg + 50 mg	1.16 ± 0.08^a^
One way analysis of variance (ANOVA)		F = 28.47 *P* < 0.001

Results are expressed as mean ± SD. One Way Analysis of Variance was used for analysis followed by Duncan's test to test the similarity between the control and three treated groups. Means with different letters indicated statistically significant difference between the compared groups in the same column.

#### Biochemical studies

**Figure 3:**
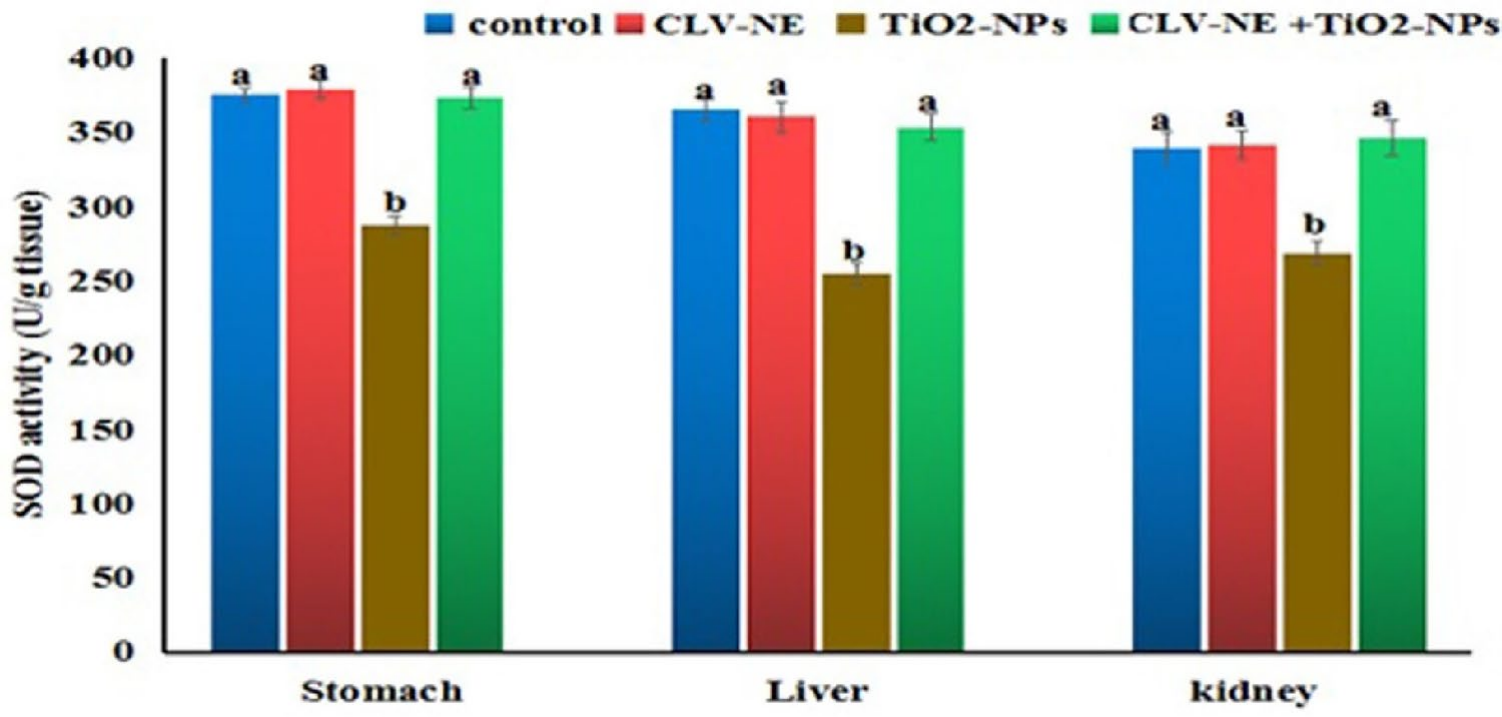
SOD activity in the stomach, liver and kidney tissue homogenate of mice in the control and various treated groups. CLV-NE = clove oil nanoemulsion, TiO_2_-NPs = titanium dioxide nanoparticles, TiO_2_-NPs + CLV-NE = titanium dioxide nanoparticles plus clove oil nanoemulsion. The data expressed as mean ± SD.

**Figure 4:**
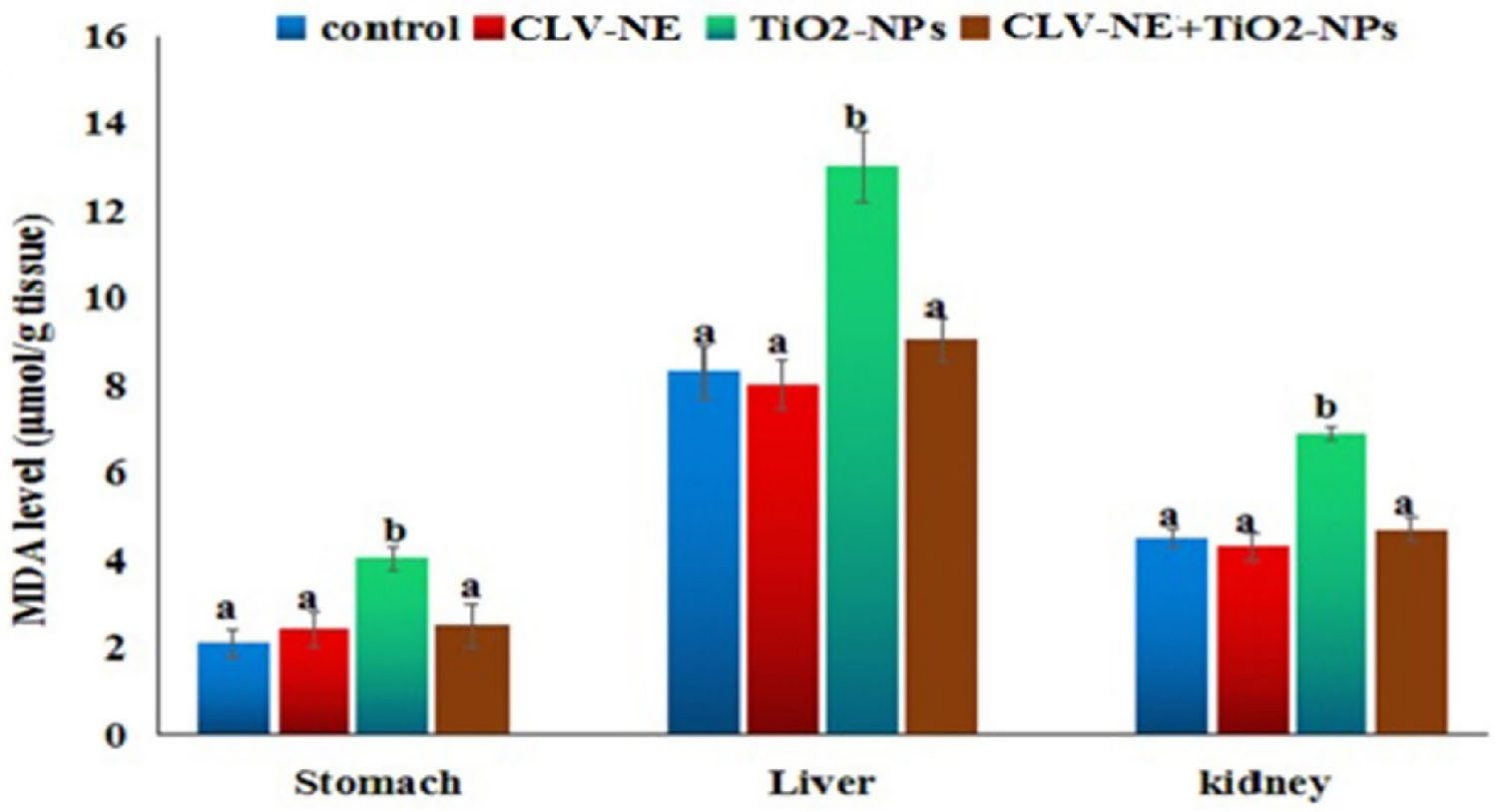
GPX activity in the stomach, liver and kidney tissue homogenate of mice in the control and various treated groups. CLV-NE = clove oil nanoemulsion, TiO_2_-NPs = titanium dioxide nanoparticles, TiO_2_-NPs + CLV-NE = titanium dioxide nanoparticles plus clove oil nanoemulsion. The data expressed as mean ± SD.

**Figure 5:**
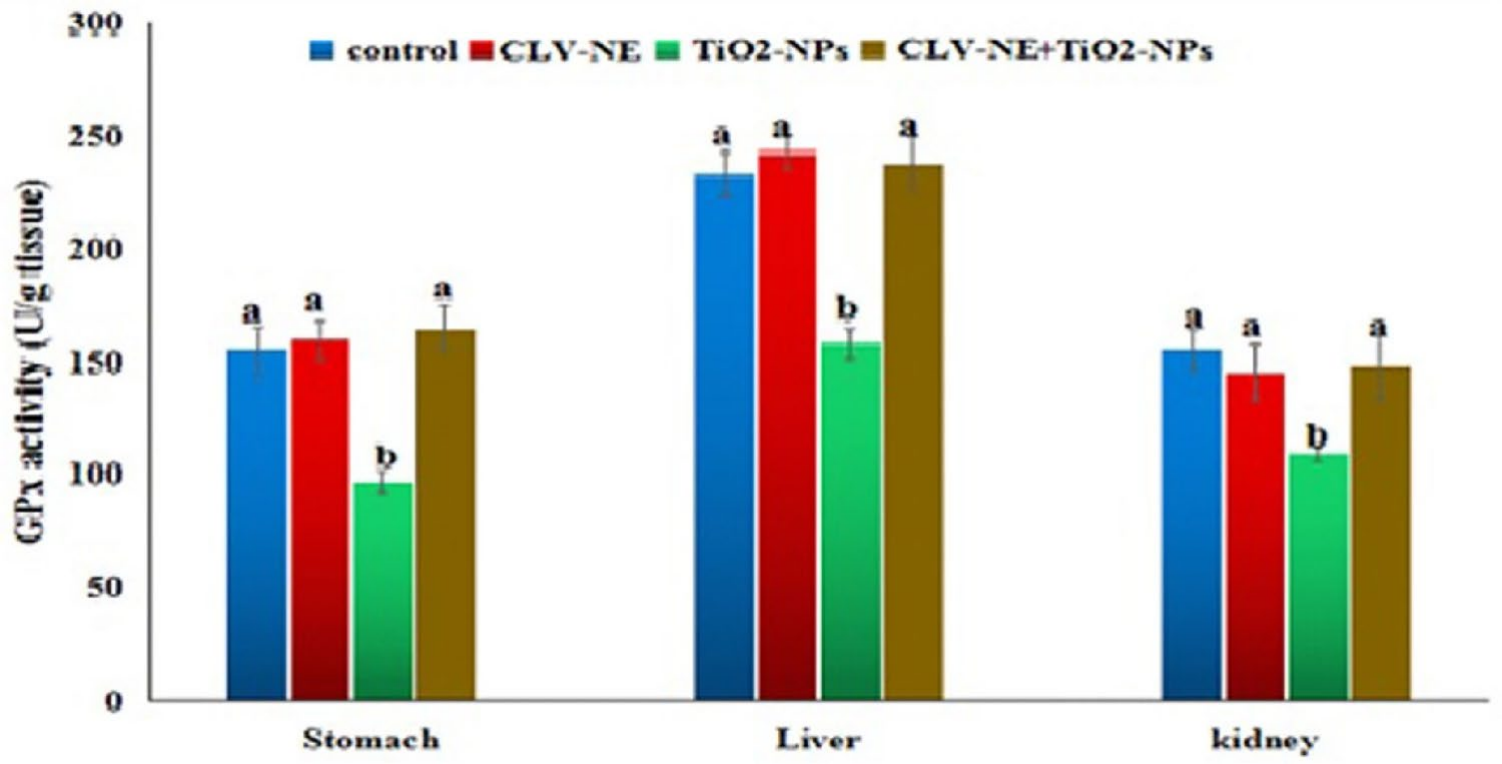
MDA levels in the stomach, liver and kidney tissue homogenate of mice in the control and various treated groups. CLV-NE = clove oil nanoemulsion, TiO_2_-NPs = titanium dioxide nanoparticles, TiO_2_-NPs + CLV-NE = titanium dioxide nanoparticles plus clove oil nanoemulsion. The data expressed as mean ± SD.

In the present study**,** the activities of superoxide dismutase (SOD) and glutathione peroxidase (Gpx) enzymes and MDA level revealed a non-significant difference between the control and CLV-NE given group in the stomach, kidney, and liver tissues as shown in Figs. 3, 4, 5. Administration of TiO_2_-NPs induced significant (*P* < 0.001) reduction in SOD and Gpx activities and significant elevation in the MDA level with respect to the control values. However, simultaneous coadministration of CLV-NE with TiO_2_-NPs significantly (*P* < 0.001) ameliorated TiO_2_-NPs induced disruption in the MDA level and activities of antioxidant SOD and GPx enzymes up to the normal control level (Figs. 3, 4, 5).

#### Histopathological examination

**Figure 6:**
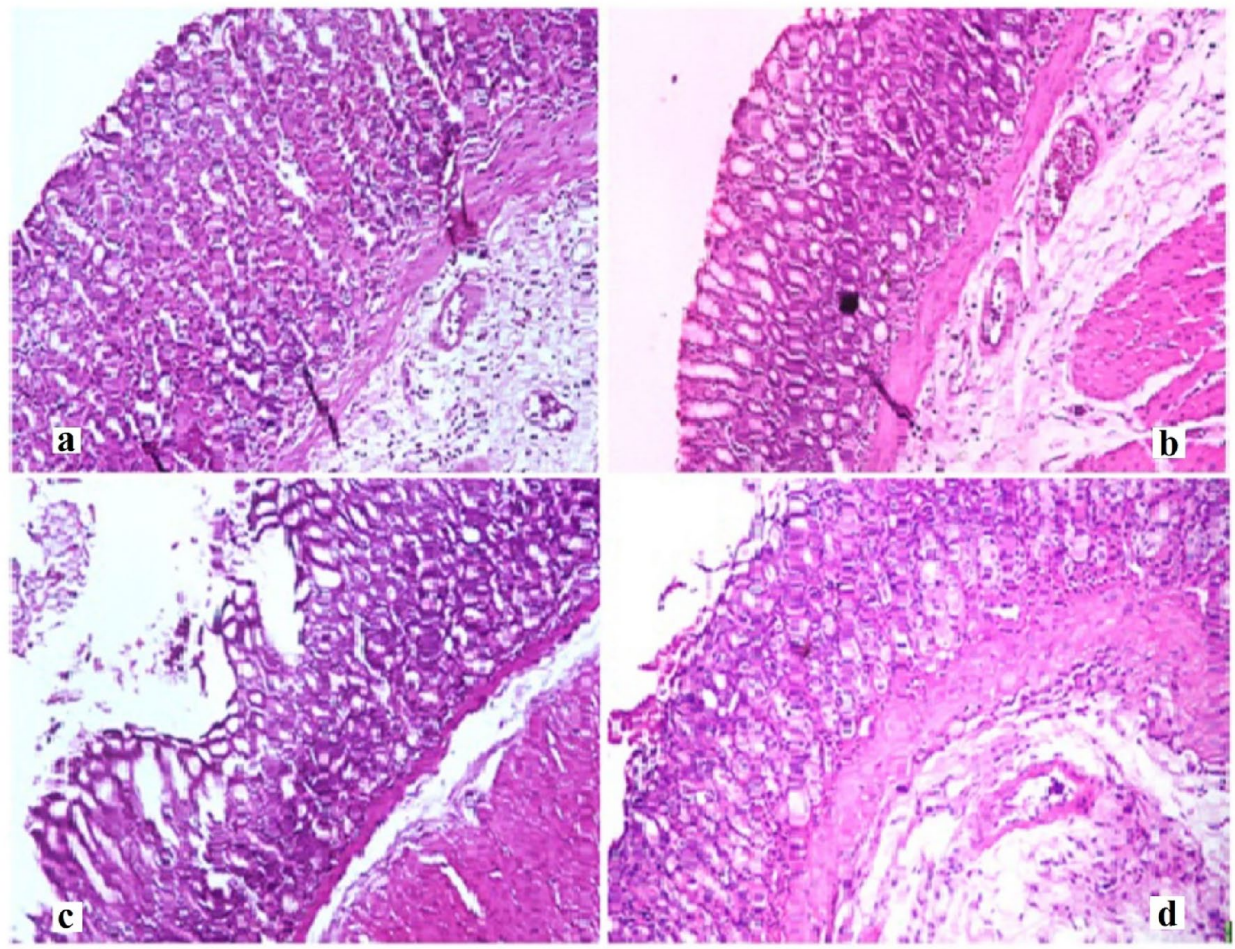
Photomicrographs of Stomach from different experimental groups stained with Hematoxylin & Eosin showing (X200). (**a**) negative control group, (**b**) clove oil nanoemulsion group, (**c**) titanium dioxide nanoparticles and (**d**) titanium dioxide nanoparticles plus clove oil nanoemulsion.

**Figure 7:**
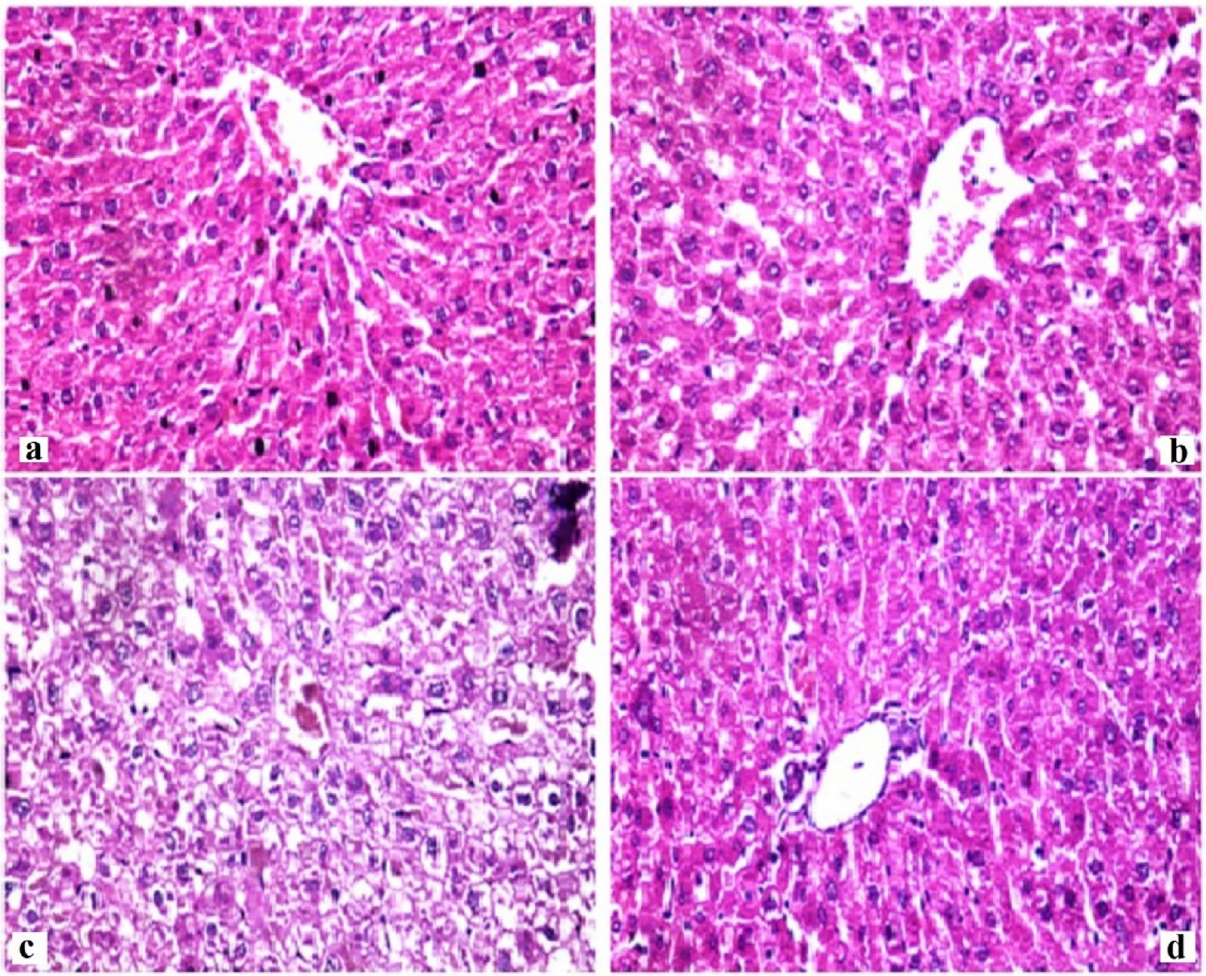
Photomicrographs of Livers from different experimental groups stained with Hematoxylin & Eosin showing (X400). (**a**) negative control group, (**b**) clove oil nanoemulsion group, (**c**) titanium dioxide nanoparticles and (d) titanium dioxide nanoparticles plus clove oil nanoemulsion.

**Figure 8:**
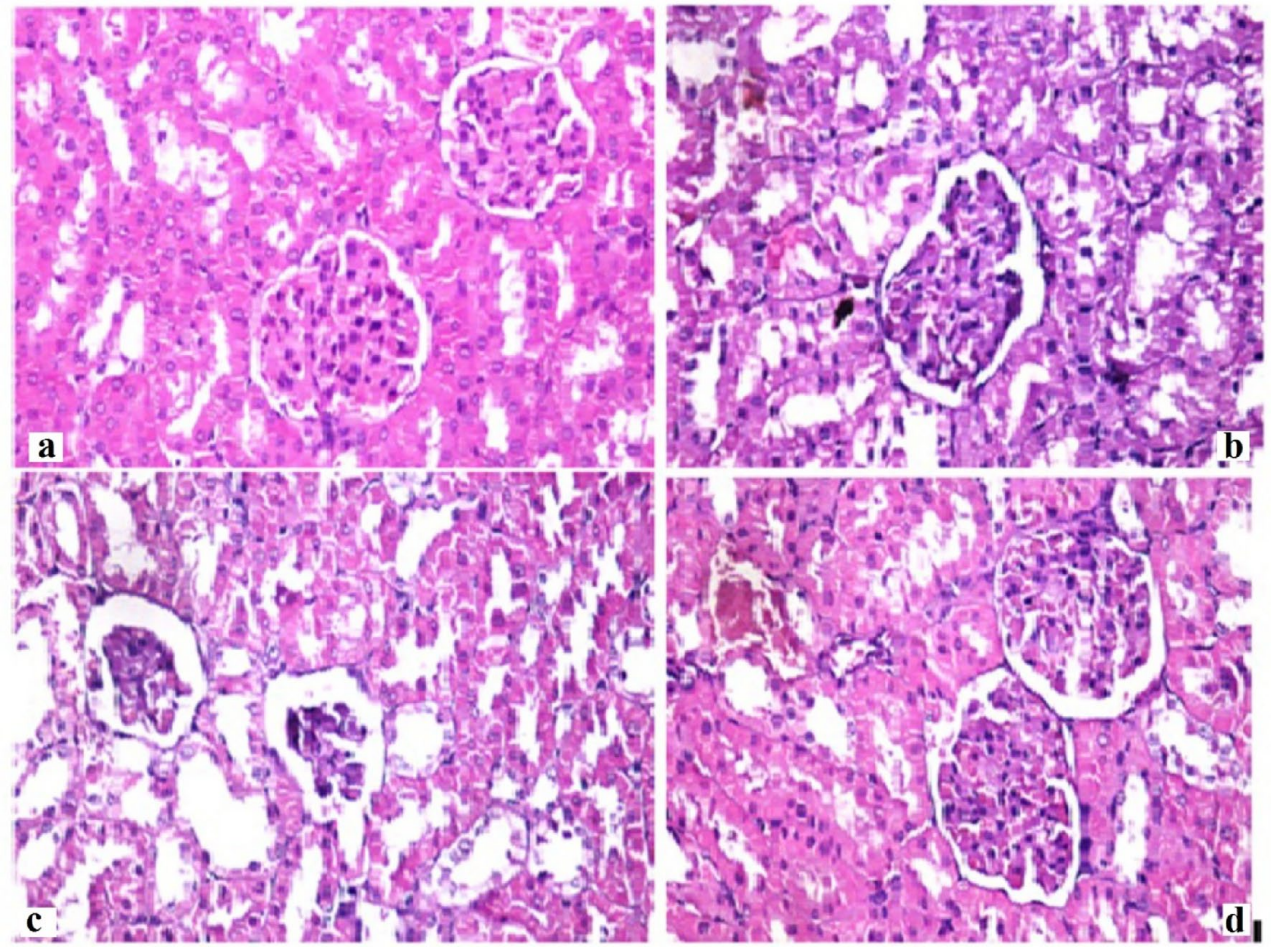
Photomicrographs of Kidneys from different experimental groups stained with Hematoxylin & Eosin (X400). (**a**) negative control group, (**b**) clove oil nanoemulsion group, (**c**) titanium dioxide nanoparticles and (**d**) titanium dioxide nanoparticles plus clove oil nanoemulsion.

The histology of stomach tissues demonstrated that control group and CLV-NE groups revealed normal stomach layers with normal mucosa, submucosa, and musculosa. However, treatment with TiO_2_-NPs caused diffused mucosal degeneration, and the desquamated mucosal epithelium, together with submucosal blood vessel congestion. While, administration of CLV-NE simultaneously with TiO_2_-NPs regenerated the mucosal layer and diminished TiO_2_-NPs induced degeneration (Fig. 6). Regarding the liver tissue, normal hepatic parenchyma with normal hepatocytes, central vein, and blood sinusoids were seen in the negative control and CLV-NE groups. Whereas TiO_2_-NPs multiple administration resulted in diffusion and degeneration of hepatocytes which were regenerated after coadministration of CLV-NE with TiO_2_-NPs (Fig. 7). For kidney tissue, Control group and CLV-NE groups showed normal renal parenchyma, healthy renal glomeruli, and renal tubules. Toxic nephrosis with the diffuse degeneration and necrosis of renal tubules were detected after the administration of TiO_2_-NPs. While coadministration of CLV-NE simultaneously with TiO_2_-NPs regenerated the renal tubules and showed no degeneration or necrosis (Fig. 8).

## Discussion

TiO_2_-NPs recently used in widespread applications in industry and medicine. However, the safety of TiO_2_-NPs exposure to stomach, liver and kidney is still unclear. CLV-NE has been proven for its effectiveness as natural antioxidant product. The present study has investigated the potential toxicity of TiO_2_-NPs in term of histological, molecular, and biochemical examinations, and the efficiency of using CLV-NE as a protective agent against TiO_2_-NPs induced toxicity.

The present data demonstrated that TiO_2_-NPs induced DNA damage and oxidative stress in the tested tissues as manifested by the significant elevations in tail length, %DNA and tail moment along with a concomitant marked reduction in the activity of antioxidant enzymes (SOD and GPX) and significant elevation in the MDA level noticed after TiO_2_-NPs. These results indicated marked DNA damage induction by TiO_2_-NPs chronic administration through a high ROS production and diminished scavenging capacity of the antioxidant enzymes. These findings agree with several authors who confirmed the induction of oxidative DNA damage due to oxidative stress after TiO_2_-NPs administration in brain, lung, Hippocampus, and kidney of mice^23,29–31^, and liver tissue of rat^32^. These studies also demonstrated that TiO_2_-NPs have elevated the induction of reactive oxygen species (ROS), and consequently increased the MDA production, which induced the oxidative DNA damage^22,33–35^. MDA destroy some functional and structural proteins leading to cell death^36^.

Moreover, ROS specially the highly cytotoxic free radical, singlet oxygen (^1^O_2_) mediates cell killing by increasing DNA damage and lipid oxidation due to its interaction with unsaturated fatty acids on the surface of the mitochondrial membrane, inducing necrosis, autophagy and apoptosis^36,37^. Abundant literature demonstrated that TiO_2_-NPs potential genotoxicity may be attributed either to their interference with the structural or functional enzymes of DNA repair system or to the direct interaction with DNA-related proteins or DNA itself^33,38^. Sun et al.^39^ stated that TiO_2_-NPs affect the proteins of endoplasmic reticulum, enter the nucleus or block its pores and then interact with DNA causing an elevation of the apoptotic related genes, oxidative stress and cytokines genes^39,40^.

Apoptosis has been detected in our study via the elevation of p53 expression level and significant DNA fragmentation after treatment with TiO_2_-NPs. The findings presented here agree with previous studies shown that TiO_2_-NPs induced elevation of apoptotic and oxidative stress genes in liver and intestine tissues in rat after 7 days of administration^41,42^. It has concluded that the large surface area of TiO_2_-NPs caused an increased production of reactive oxygen species which resulted in imbalanced homeostasis between oxidation and anti-oxidative activities that implicated with the oxidative stress, genotoxicity and cytotoxicity^43^.

The increased production of ROS after administration of TiO_2_-NPs may be rationalized by activation of heme oxygenase 1 through p38-Nrf-2 signaling pathway or attachment of TiO_2_-NPs to prion protein the plasma membrane protein which distorts prion signaling function. This prompts the activation of NADPH oxidase via prion-dependent pathway and consequently increases the production of ROS that changes redox equilibrium^44,45^.

Further, cytoplasmic ROS are known to induce pathophysiological diseases in several tissues, which have indicated in this study by the abnormal architecture noticed in the stomach, liver and kidney tissues of mice orally given TiO_2_-NPs alone. These results may be attributed to the high production of ROS, inflammation, and finally cell death due to death of mitochondria^46^. Beside the ordinary way of ROS production, accumulation of mitochondrial ROS due to impairment of its scavenger mechanism leads to organelle dysfunction and cell death^36^. Taken together, TiO_2_-NPs can induce toxicity by three mechanisms; by ROS induction, or lipid peroxidation of the cell membrane and its damaged due to attachment of nanoparticles on the cell surface by electrostatic force, or by TiO_2_-NP attachment to intracellular organelles like mitochondria after cell membrane destruction^10^.

On the other hand, co-administration of CLV-NE, a powerful antioxidant natural product mitigated TiO_2_-NPs induced DNA damage and oxidative stress along with restoring the normal tissues' structure. This ameliorative effect of CLV-NE was demonstrated by the remarkable reductions in the DNA damage level and MDA level along with significant increases in the activity of antioxidant SOD and Gpx enzymes, and may be explained by its high content of anti-inflammatory and antioxidant eugenol derivative compounds that prevent prostaglandin synthesis and chemotaxis of neutrophil^47,48^.

In addition, eugenol has an effective inhibitory effect on protein denaturation that may stabilize the cell membrane and inhibiting the seepage of intracellular enzymes^49^. More importantly, eugenol reported as an effective antioxidant therapeutic agent that could decrease the ROS in cardiovascular disease by increasing the thioredoxin reductase activity^50^. Decreasing the oxidative stress restore the scavenging activities of SOD and GPX which in turn abolishes the oxidative DNA damage and histopathological injuries induced by TiO_2_-NPs. abolishment of TiO_2_-NPs induced tissues injuries was detected in this study by the noticed regeneration and restoration of normal tissues' architecture after coadministration of CLV-NE with TiO_2_-NPs In addition, emulsification extended the clove oil’ scavenging time of free radicals due to increased surface area^51^. According to polar paradox theory, high efficiency of antioxidant activity of clove oil emulsion is due to its non-polarity in O/W emulsion^51,52^.

## Conclusion

In conclusion, administration of TiO_2_-NPs induced oxidative DNA damage with increased ROS production that overwhelmed the capacity of SOD and GPX and consequently resulted in elevated level of MDA, all led to apoptosis induction and histopathological changes in stomach, liver, and kidney. However, Concurrent administration of CLV-NE with TiO_2_-NPs sustained the genomic DNA integrity and normal tissues architecture through its free radicals scavenging capacity that overcome MDA production restore the activity of antioxidant enzymes to normal level. Further research is still needed to explore the efficiency of using CLV-NE as a protective or even a therapeutic agent to manage the toxic effects of TiO_2_-NPs use for chronic period.

### Supplementary Information

Supplementary Information.

## Data Availability

The datasets used and/or analyzed during the current study are available from the corresponding author on reasonable request.
