# Author Correction: Modulation efficiency of clove oil nano-emulsion against genotoxic, oxidative stress, and histological injuries induced via titanium dioxide nanoparticles in mice

**DOI:** 10.1038/s41598-024-63602-x

**Published:** 2024-06-05

**Authors:** Hanan R. H. Mohamed, Sawsan El-Shamy, Sherein S. Abdelgayed, Rofida Albash, Haidan El-Shorbagy

**Affiliations:** 1 Zoology Department, Faculty of Science, Cairo University, Giza, Egypt; 2 Faculty of Biotechnology, October University for Modern Science and Arts, 6th October, Giza Egypt; 3 Pathology Department, Faculty of Veterinary Medicine Cairo University Giza, Giza, Egypt; 4 College of Oral and Dental Surgery, Misr University for Science and Technology, 6th of October, Giza Egypt; 5 Department of Pharmaceutics, Faculty of Pharmaceutical Sciences, Misr University for Science and Technology, 6th of October, Giza Egypt

Correction to: *Scientific Reports* 10.1038/s41598-024-57728-1, published online 02 April 2024

In the original version of this Article Rofida Albash was incorrectly affiliated with ‘College of Oral and Dental Surgery, Misr University for Science and Technology, 6th of October, Giza, Egypt’.

The correct affiliation is listed below.

Department of Pharmaceutics, Faculty of Pharmaceutical Sciences, Misr University for Science and Technology, 6th of October, Giza, Egypt.

The original Article has been corrected.

