# Supplementary figures and images for: Modulation efficiency of clove oil nano-emulsion against genotoxic, oxidative stress, and histological injuries induced via titanium dioxide nanoparticles in mice
Source: Sci Rep. 2024 Apr 2;14:7715. doi: 10.1038/s41598-024-57728-1 (PMC10987579; doi:10.1038/s41598-024-57728-1)

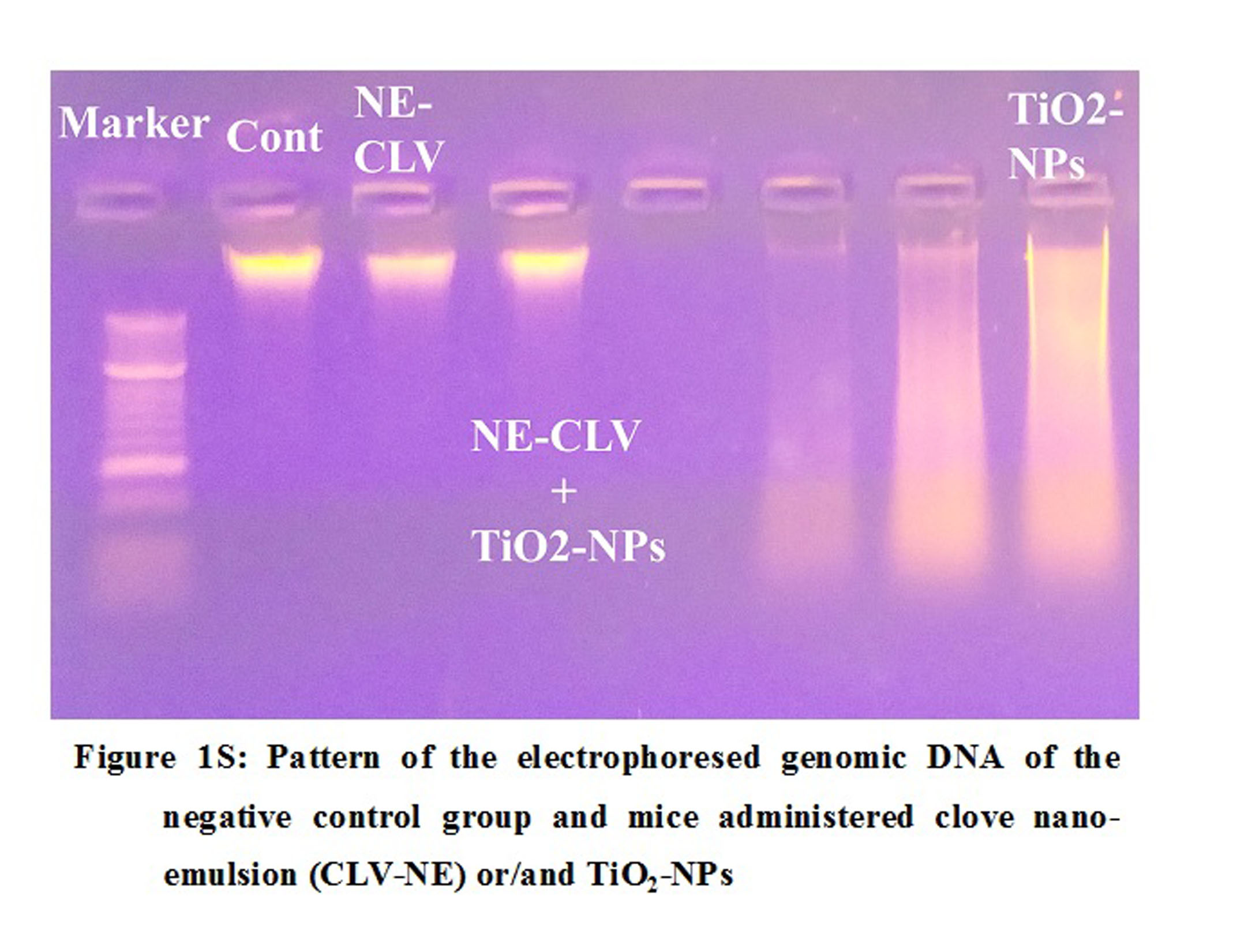

Supplement: Supplementary file 1 — Supplementary Information. [file 41598_2024_57728_MOESM1_ESM.jpg]
